# Biological Functions of the Intrinsically Disordered N-Terminal Domain of the Prion Protein: A Possible Role of Liquid–Liquid Phase Separation

**DOI:** 10.3390/biom11081201

**Published:** 2021-08-12

**Authors:** Stella A. Polido, Janine Kamps, Jörg Tatzelt

**Affiliations:** 1 Department Biochemistry of Neurodegenerative Diseases, Institute of Biochemistry and Pathobiochemistry, Ruhr University Bochum, 44801 Bochum, Germany; stella.polido@rub.de (S.A.P.); janine.kamps@rub.de (J.K.); 2 Cluster of Excellence RESOLV, Ruhr University Bochum, 44801 Bochum, Germany

**Keywords:** prion, intrinsically disordered, stress protection, liquid–liquid phase separation, neurodegeneration

## Abstract

The mammalian prion protein (PrP^C^) is composed of a large intrinsically disordered N-terminal and a structured C-terminal domain, containing three alpha-helical regions and a short, two-stranded beta-sheet. Traditionally, the activity of a protein was linked to the ability of the polypeptide chain to adopt a stable secondary/tertiary structure. This concept has been extended when it became evident that intrinsically disordered domains (IDDs) can participate in a broad range of defined physiological activities and play a major functional role in several protein classes including transcription factors, scaffold proteins, and signaling molecules. This ability of IDDs to engage in a variety of supramolecular complexes may explain the large number of PrP^C^-interacting proteins described. Here, we summarize diverse physiological and pathophysiological activities that have been described for the unstructured N-terminal domain of PrP^C^. In particular, we focus on subdomains that have been conserved in evolution.

## 1. The Prion Protein

The mammalian prion protein (PrP) was first identified as a protease-resistant protein in brain extracts, which co-purified with the infectious scrapie agent [1]. The identification of the corresponding gene revealed that PrP is a constitutively expressed host protein, mainly found in neuronal and immune cells [2,3]. From these and subsequent studies, the concept emerged that the disease-causing mechanism in mammalian prion diseases is a conformational transition of the cellular isoform of PrP (PrP^C^) into PrP^Sc^, an aberrantly folded conformer with neurotoxic and infectious properties [4]. The central role of PrP^C^ in the formation of PrP^Sc^ and infectious prions is highlighted in PrP^C^-deficient mice and goats, which are resistant to prion infection [5,6]. Biogenesis of PrP^C^ is characterized by a series of co- and posttranslational modifications (see [7]). After import into the endoplasmic reticulum (ER), PrP is modified in the secretory pathway with two N-linked glycans of complex structure [8,9,10,11] and a C-terminal glycosylphosphatidylinositol (GPI) anchor [12], which targets mature PrP^C^ to the outer leaflet of the plasma membrane. Conversion into PrP^Sc^ is thought to occur after mature PrP^C^ has reached the plasma membrane or is re-internalized for degradation [13]. However, neither the two N-linked glycans [14,15] nor the GPI anchor of PrP^C^ [16,17] are essential for the formation of infectious prions.

## 2. The Intrinsically Disordered N-Terminal Domain of PrP

**Figure 1:**
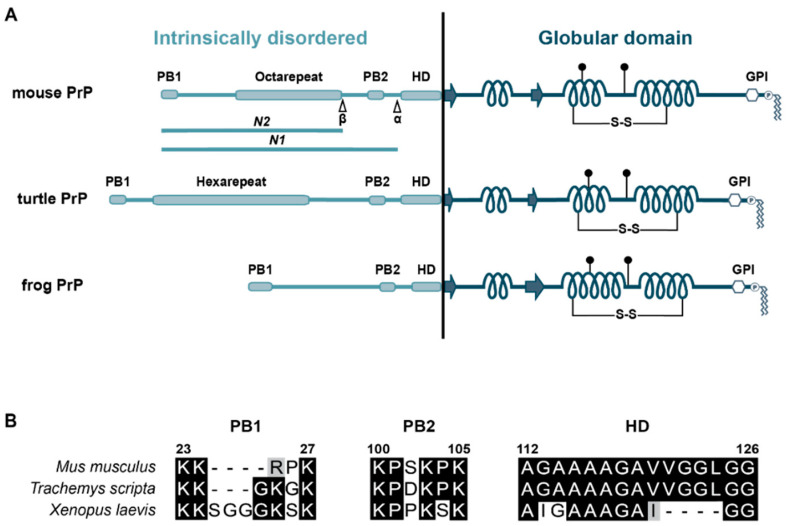
(**A**) Schematic representation of PrP^C^ from mouse, turtle, and frog. PB: polybasic motif; β: cleavage site that generates N2; α: cleavage site that generates N1; HD: hydrophobic domain; arrows: β-strand; coils: α-helices; S-S: disulfide bridge; filled circles: N-linked glycans; GPI: glycosylphosphatidylinositol anchor. (**B**) Sequence alignments of PB1, PB2, and the HD. Identical residues are marked in black, similar residues in gray (GenBank accession numbers: M18070.1, XP_034617687.1, AAH94089.1). The numbering of the residues refers to mouse PrP^C^.

The first published structure of mouse PrP^C^ revealed the presence of a highly structured C-terminal domain of some 100 amino acids with three alpha-helical domains and two short beta-strands [18]. Interestingly, the remaining N-terminal domain of similar length proved to be completely intrinsically disordered (Figure 1A) [19,20]. This modular structure of PrP^C^ is conserved through evolution; moreover, the C-terminal domain of human (amino acid (aa) 121–230), chicken (aa 121–225), turtle (aa 121–225), and xenopus (aa 90–222) PrP^C^ show extensive structural similarities [21] (Figure 1A). While the N-terminal unstructured domain shows considerable sequence diversity between tetrapod classes (Figure 1), it contains three highly conserved regions: (1) an internal hydrophobic domain (HD) first described as a putative transmembrane domain [22], (2) a stretch of positively charged amino acids distal to the ER signal peptide (PB1), and (3) a stretch of positively charged amino acids at the C-terminal end of the unstructured domain (PB2) (Figure 1B). In this review, we discuss in detail that these regions are associated with some physiological and pathophysiological activities described for PrP^C^. However, the N-terminal domain is not only important in the context of full-length PrP^C^. A considerable fraction of mature PrP^C^ is proteolytically processed in vivo, resulting in the release of two distinct soluble, unstructured N-terminal fragments. One fragment, designated N1, is formed by α-cleavage of PrP^C^ under physiological conditions approximately at amino acid position 110. A second cleavage around amino acid position 90 (β-cleavage) is mainly observed under pathological conditions and liberates the fragment N2 [23,24,25,26,27,28] (Figure 1A). These findings raise the intriguing question of whether PrP^C^ processing plays a role in generating soluble N-terminal fragments with distinct biological activities (see [26,29,30]).

## 3. Physiological and Pathophysiological Activities of the N-Terminal Domain 

The major phenotype of PrP^C^-deficient mice and goats is their resistance to prion diseases [5,6]. Apart from that, a number of biological activities have been attributed to PrP^C^, such as modulation of synaptic transmission and neuronal excitability; protection against oxidative stress; neuroprotective and neurotoxic signaling; and a role in cell differentiation, neuronal adhesion, and neuro-immune crosstalk. In the context of this review, we focus specifically on biological activities of PrP that are dependent on, or are modulated by, the N-terminal domain or conserved regions therein. For an overview of activities that have been ascribed to PrP thus far, we would like to refer the reader to previous comprehensive reviews [31,32,33,34,35,36,37,38,39,40,41,42,43].

### 3.1. The N-Terminal Domain and Prion Diseases

**Figure 2:**
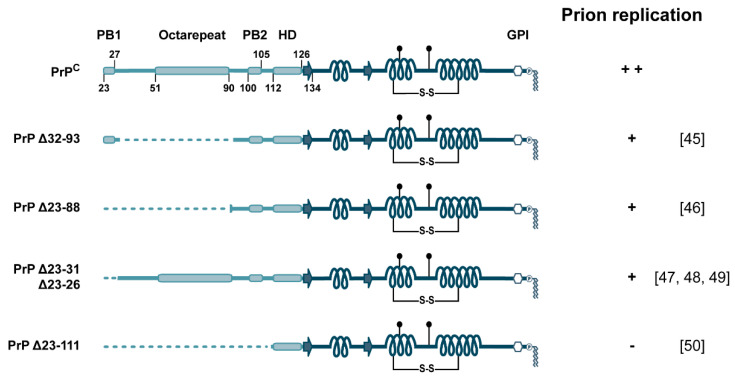
The role of the N-terminal domain in prion propagation. Schematic representation of PrP^C^ variants and their ability to support replication of infectious prions in mice after inoculation with scrapie prions. The numbering of the residues refers to mouse PrP^C^. Respective publications are in brackets.

The prion protein identified in proteinase K-treated brain extracts of scrapie-infected hamsters was PrP27–30, the protease K-resistant core of PrP^Sc^ that lacks aa 23–90. PrP27–30 transmits prion disease, indicating that the infectious properties of prions are not dependent on the N-terminal part of PrP comprising N2 [1,44]. This part is also dispensable for the conversion of PrP^C^ into PrP^Sc^, since propagation of infectious prions is supported in transgenic mice expressing only a truncated variant of PrP^C^ devoid of aa 32–93 [45] or aa 23–88 [46]. However, incubation time was significantly increased in these mice, revealing that the unstructured domain of PrP^C^ modulates propagation of prions. Notably, mice expressing a variant of PrP^C^ deleted for only aa 23–31 already display a dramatically reduced susceptibility to prion infection and accumulate significantly reduced levels of PrP^Sc^. This study highlights an important role of the PB1 domain of PrP^C^ in the conversion into PrP^Sc^ [47], a finding corroborated later [48,49]. On the other hand, the N2 domain (aa 23–90) or regions therein only modulate prion propagation; deleting the N1 domain prevents prion disease. Transgenic mice expressing PrP^C^(Δ23–111) remain healthy after inoculation with scrapie prions and do not accumulate protease-resistant PrP^Sc^ [50] (Figure 2).

Inherited prion diseases in humans are caused by mutations in the PrP gene (*PRNP*). Excluding the octapeptide repeat insertions, all pathogenic mutations found in the N-terminal unstructured domain of PrP^C^ are distal to amino acid 90 [51]. Interestingly, a recent high-resolution structure obtained by cryo-EM showed that the section containing residues 95–112, which is unstructured in PrP^C^, contains two beta-strands upon conversion into PrP^Sc^ [52].

### 3.2. The N-Terminal Domain and Neurotoxicity

In this section, we would like to illustrate by means of two examples how the N-terminal domain is involved in neurotoxic signaling of PrP^C^ independently of the formation of infectious prions: first, by deleting the internal highly conserved hydrophobic domain, and second, by interacting with neurotoxic protein assemblies.

**Figure 3:**
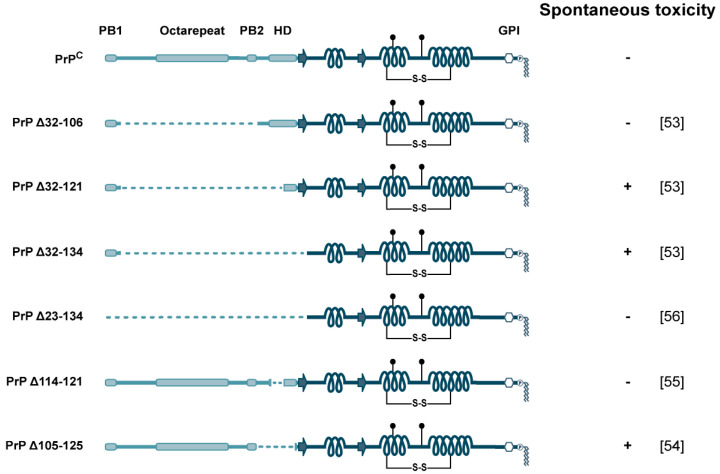
Neurotoxic activity of PrP^C^ variants with N-terminal deletions. Schematic representation of PrP^C^ variants and their spontaneous activity to induce neuronal dysfunction in transgenic mice. Respective publications are in brackets.

From a study designed to identify regions of PrP^C^ involved in the formation of infectious prions, it emerged that deletions in the internal HD can convert PrP^C^ into a neurotoxic protein that is not infectious. Transgenic mice expressing PrP^C^ with a deletion of residues 32–80, 32–90, or 32–106 showed no overt phenotype. However, severe ataxia and neuronal cell death was observed in transgenic mice expressing PrP^C^ (Δ32–121) or PrP^C^ (Δ32–134) [53]. Notably, the deletion of 20 amino acids of the HD (Δ105–125) is sufficient to create a neurotoxic PrP variant [54], while a shorter deletion (Δ114–121) is not [55]. Although the relevance of the mechanisms underlying neurotoxic activity to the pathogenesis of prion diseases remains unclear, there are some noteworthy features of PrPΔHD. First, while expression of PrPΔHD causes neurodegeneration, it does not form infectious prions. Second, co-expression of wild-type PrP^C^ suppresses the neurotoxic activity of PrPΔHD [53,54,55]. Third, transgenic mice expressing PrP^C^ (Δ23–134) instead of PrP^C^ (Δ32–134) display no clinical symptoms or neuropathology, indicating that PB1 is required for the toxic phenotype of PrP^C^ (Δ32–134) [56] (Figure 3).

The first hints that wild-type GPI-anchored PrP^C^ could serve as a toxic receptor of pathogenic protein conformers emerged from an elegant study by Brandner and colleagues who grafted neural tissues overexpressing PrP^C^ into the brains of PrP^0/0^ mice. After infection with scrapie prions, the PrP^C^-expressing graft propagated PrP^Sc^ and developed histopathological alterations characteristic of scrapie disease. However, the surrounding PrP^C^-deficient tissue remained healthy, despite the accumulation of PrP^Sc^ [57]. Using a cell culture model, we provided further evidence that PrP^Sc^ can induce neurotoxic signaling via an interaction with PrP^C^ at the plasma membrane. Importantly, the unstructured N-terminal domain of PrP^Sc^ was required for this activity [58]. A series of subsequent studies then revealed that the ability of PrP^C^ to relay toxic signals after binding to misfolded protein assemblies is of broad pathological significance. In a landmark study, the Strittmatter group showed that toxicity of oligomeric Aβ can be mediated by PrP^C^ [59]. Moreover, PB1 and PB2 in the unstructured N-terminal domain of PrP^C^ were mapped as the Aβ-binding sites [59,60]. While a possible pathological role of PrP^C^ as a receptor of Aβ was initially discussed critically, numerous studies in cultured cells and transgenic mice have convincingly supported this concept (see [39,61]). Consistent with the ability of intrinsically disordered domains to interact with different substrates independently of their primary sequence, we then observed that completely unrelated beta-sheet rich oligomeric assemblies; for example, those formed by the yeast prion protein Sup35 or an artificial beta-sheet peptide can bind to the N-terminal domain of PrP^C^ and induce cell death [62]. Finally, neurotoxic signaling of soluble α-synuclein and Tau assemblies via binding to the N-terminal domain of PrP^C^ was demonstrated [63,64,65]. It will now be interesting to explore the intracellular signaling pathways activated by the PrP^C^/oligomer complex in more detail and harness them in developing therapeutic strategies for neurodegenerative diseases.

### 3.3. The N-Terminal Domain and Neuroprotection: A Role of Soluble Fragments

From the analysis of PrP^C^ variants devoid of the N-terminal domain or subdomains thereof, it became apparent that some of the biological activities of full-length GPI-anchored PrP^C^ are dependent on its N-terminal regions. We now turn to intriguing findings showing that soluble N-terminal fragments of PrP^C^ have biological activities independently of the globular C-terminal domain. Notably, analyzing the brain interactome of soluble N1 revealed that the intrinsically disordered N-terminal domain is a major mediator of PrP interactions [66].

It was previously shown that the stress protective activity of full-length PrP requires the N-terminal domain [58,67,68]. Interestingly, this activity seems to be at least partially independent of the C-terminal domain and/or GPI-anchoring. By employing recombinant proteins added to cultured cells, different groups presented evidence that soluble N1 and N2 can protect against various stress paradigms [69,70,71,72]. Notably, in one study [69] N1, but not N2, displayed neuroprotective activity in vivo and in vitro by modulating the p53 signaling pathway. This finding may indicate that PB2, which is missing in N2, might be important for a biological activity specific for N1.

Another interesting example for soluble N-terminal fragments showing an activity initially ascribed to GPI-anchored full-length PrP emerged from the observation that the loss of PrP^C^ in mice leads to a chronic demyelinating polyneuropathy affecting Schwann cells [73], a phenotype that was corroborated later in PrP^C^-deficient goats [74]. In a follow-up study in mice, it was then demonstrated that a soluble N-terminal fragment has the same activity as full-length PrP^C^ in activating the G protein-coupled receptor Adgrg6. Intriguingly, this activity was dependent on PB1: substitution of the cationic residues in PB1 by alanines abolished the activity of the N-terminal fragment to activate Adgrg6 [75]. 

The first observation that a soluble PrP fragment can protect against neurotoxicity induced by pathogenic protein assemblies was made in transgenic mice expressing a secreted full-length PrP-immunoglobulin Fc (PrP-Fc_2_) fusion protein. After inoculation with prions, PrP-Fc_2_ was not converted into PrP^Sc^, and the onset of prion disease was delayed [76]. A plausible mode of action would be that secreted PrP-Fc_2_ interacts with PrP^Sc^ and thereby interferes with its toxic signaling via GPI-anchored PrP^C^.

**Figure 4:**
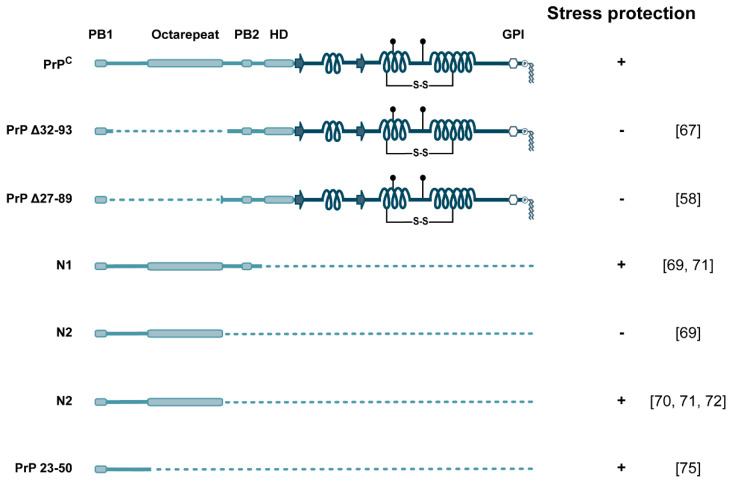
Neuroprotective activity of PrP. Schematic representation of PrP^C^ variants and their stress protective activity in cell culture and transgenic mouse models. Respective publications are in brackets.

On the basis of the role of the N-terminal domain in mediating the interaction of PrP^C^ with Aβ, we were wondering whether a soluble N1 fragment would protect against Aβ-induced toxicity. Notably, the isolated N-terminal domain of PrP cannot be expressed as a secreted protein in mammalian cells or neurons of transgenic mice, since ER import of such a C-terminally truncated PrP construct is significantly impaired [77,78,79]. We therefore employed a fusion protein composed of N1 and the Fc portion of human IgG_1_. Indeed, the secreted N1 fragment efficiently bound to Aβ and significantly reduced its toxic signaling via PrP^C^ [62]. Further experimental evidence for a protective activity of the soluble N1 fragment against toxic effects of oligomeric Aβ was provided subsequently in different model systems, including primary neurons, *Caenorhabditis elegans*, and mouse models of Aβ-induced memory dysfunction [80,81,82,83] (Figure 4).

These two examples revealed a biological function of soluble N-terminal fragments of PrP^C^ at the outer leaflet of the plasma membrane or in the extracellular space. What is missing thus far is experimental evidence that soluble N-terminal fragments have a biological function in intracellular compartments. To date, it was shown that the N-terminal domain has the capacity to enter cells. PB1 and PB2 have similarities with cell-penetrating peptides and the fusion of PB1 or PB2 to heterologous proteins promote their cellular uptake and delivery into the cytoplasm [84,85].

## 4. The N-Terminal Domain Is Necessary and Sufficient for Liquid–Liquid Phase Separation of PrP

Multiple cellular processes, including receptor-mediated signaling and formation of stress granules, are coordinated by biomolecular condensates or membrane-less compartments that can form via liquid–liquid phase separation (LLPS) (see [86,87,88,89]). Furthermore, several proteins implicated in neurodegenerative diseases have been shown to undergo LLPS, leading to a concept that altered LLPS can promote the formation of protein assemblies with neurotoxic properties (see [90,91,92,93]).

Indeed, LLPS of full-length PrP has been described recently [94,95,96,97,98]; however, the molecular mechanisms underlying the formation of PrP-containing liquid droplets remain unknown. 

In a recent study, we provided insight into the mechanism underlying the propensity of the mammalian prion protein to undergo LLPS [99]. Our study revealed that the intrinsically disordered N1 fragment of PrP is necessary and sufficient for the formation of biomolecular condensates, emphasizing the concept that intrinsically disordered and low-complexity regions are important drivers of phase separation [100]. Furthermore, a mutational analysis revealed that LLPS of N1 is governed at the molecular level mainly by intermolecular cation–π interactions of the positively charged residues in PB1 and PB2 with aromatic side chains [99]. 

Although there are only few publications to date that have examined LLPS of PrP in detail [94,95,96,97,98,99], they provided experimental evidence that LLPS could play a role in regulating (patho)physiological activities of PrP and formation of infectious prions. Consistent with the concept that biomolecular condensates can be precursors of pathogenic protein aggregates, it was shown that after LLPS of recombinant full-length PrP, a rapid liquid–solid phase transition occurred, leading to the formation of β-sheet-rich and PK-resistant amyloid [94]. Notably, the unstructured N-terminal domain of PrP was required for the initial LLPS, supporting our finding that LLPS of PrP-C1 and -C2 is impaired [99]. Interestingly, it was already described some time ago that large globular protein assemblies preceded the formation of PrP145X amyloid fibrils in vitro. While it was not shown experimentally, the images of the globular structures are indicative of biomolecular condensates formed by PrP145X via LLPS [101]. Another intriguing observation is that binding of neurotoxic Aβ oligomers to the polybasic motifs converts liquid-like droplets of full length PrP into hydrogel and induces a conformation change of PrP [97,102], suggesting that aberrant phase transition of PrP^C^ may be associated with the activity of the PrP/Aβ complex to induce neurotoxic signaling [97]. Thus far, a possible role of LLPS in the neuroprotective activities of full length PrP or the liberated N-terminal fragments has only been indirectly demonstrated: PrP variants that lack a stress protective activity in cell culture and animal models [58,67] failed to undergo LLPS in vitro [94,99]. Vice versa, N1 formed biomolecular condensates in vitro [99] and showed stress-protective activity in cell culture models [69,71]. It will now be interesting to analyze LLPS of PrP^C^ and its proteolytic fragments in a cellular context and, in particular, to address the role of membrane anchoring via the GPI-anchor.
